# Functionalization of OMVs for Biocatalytic Applications

**DOI:** 10.3390/membranes13050459

**Published:** 2023-04-24

**Authors:** Lita Amalia, Shen-Long Tsai

**Affiliations:** Department of Chemical Engineering, National Taiwan University of Science and Technology, Taipei City 10607, Taiwan

**Keywords:** vesicle, functionalization, immobilization, enzyme, catalyst

## Abstract

Outer membrane vesicles (OMVs) are miniature versions of gram-negative bacteria that contain almost the same content as their parent cells, particularly in terms of membrane composition. Using OMVs as biocatalysts is a promising approach due to their potential benefits, including their ability to be handled similarly to bacteria while lacking potentially pathogenic organisms. To employ OMVs as biocatalysts, they must be functionalized with immobilized enzymes to the OMV platform. Various enzyme immobilization techniques are available, including surface display and encapsulation, each with advantages and disadvantages depending on the objectives. This review provides a concise yet comprehensive overview of these immobilization techniques and their applications in utilizing OMVs as biocatalysts. Specifically, we discuss the use of OMVs in catalyzing the conversion of chemical compounds, their role in polymer degradation, and their performance in bioremediation.

## 1. Introduction

**Figure 1:**
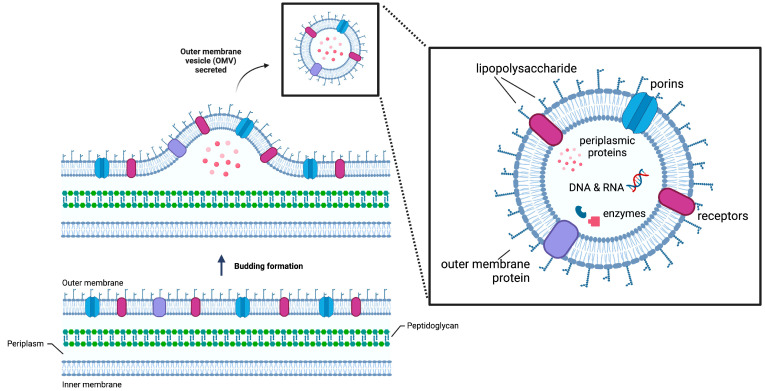
The vesiculation mechanism in gram-negative bacteria is not a passive process [1,2]. It begins with the formation of a bud, followed by the discharge of a spherical material that encapsulates the periplasmic contents within the vesicle’s lumen. The image in the box shows details of the typical composition of OMVs [4]. (Created with BioRender.com).

Outer membrane vesicles (OMVs) are intriguing and versatile extracellular vesicles that have garnered increasing attention in recent years. These nano-sized, spherical proteoliposomes are continuously discharged from the outer membrane of gram-negative bacteria via a vesiculation mechanism that involves the separation of the outer membrane from the underlying peptidoglycan layer (Figure 1). There are several theories regarding this mechanism; the most commonly reported is that vesiculation involves the relocation of defective covalent bonds between the outer membrane and the peptidoglycan layer. Consequently, the outer membrane grows faster than the layers beneath it, allowing it to protrude into a budding site and resulting in OMVs [1,2]. Once the OMVs disengage from the bacterial surface, they range in size from 20–300 nm in diameter [3]. They partially encase the periplasmic contents with a phospholipid membrane, making them miniature versions of bacteria.

OMVs are more than just lipid bilayer structures; they contain outer membrane proteins (OMPs), a thin layer of peptidoglycan, lipopolysaccharides (LPSs), and other periplasmic constituents that give them a complex composition [4]. This composition is highly dependent on the type of bacterial strain from which the OMVs are derived and can be tailored to suit a wide range of biotechnological applications. Moreover, OMVs are highly attractive for biotechnological applications because they contain essential outer membrane-derived components, such as porins and proteins, for transporting micro and macro elements, making them highly effective for biocatalysts [3,4,5,6,7]. In addition, OMVs have potential applications in drug delivery, vaccines, and as a source of antigens for diagnostic assays. Given their versatility and potential applications, OMVs are an exciting area of research with a promising future [3,4,5].

OMVs can transport a diverse range of biomolecules, such as nucleic acids, proteins, and lipids, over extended distances in their lumen. Due to these properties, OMVs play an essential role in intercellular communication and are also involved in forming biofilms [1,8,9,10]. In addition, OMVs have been found to enhance bacterial survival in various ways, including reducing levels of toxic compounds, neutralizing antimicrobial peptides that influence antibiotic resistance, and releasing misfolded proteins from cells to reduce cell stress [1,11,12,13]. Moreover, OMVs can serve as suitable containers for encapsulating biomolecules, making them promising for biomedical applications, such as drug delivery, vaccine administration, and use as therapeutic agents [1,3]. Indeed, the most common and well-known application of OMVs is in developing vaccines. This is due to the fact that OMVs contain antigen components that are useful for vaccine formulation—specifically LPS, which also serves as an adjuvant to induce immune response [9]. However, scientists are also exploring other potential uses of OMVs in biotechnology.

Numerous studies have employed protein translocation mechanisms from the cytoplasm and periplasm to display enzymes on cell surfaces [14,15], which are among the enzyme-immobilization methods. As miniature replicas of bacterial cells with the same membrane structure as their progenitors, OMVs offer the same potential as that of bacterial outer membranes for immobilizing enzymes. Compared to cell-surface display methods, OMVs are smaller than bacterial cells and lack organelles, thereby providing alternative immobilization techniques, such as entrapment or encapsulation, to enhance enzyme stability [16,17,18,19]. Consequently, OMVs functionalized with enzymes can be utilized as nanobioreactors for bioconversion and other applications, such as bioremediation. This paper aims to review the use of OMVs as biocatalysts by focusing on the functionalization of OMVs via various immobilization techniques. In addition, the literature describes the application of modified OMV-enzymes and provides an overview of recent progress, highlighting the potential for future development of OMVs as biocatalysts.

## 2. OMVs Functionalization by Immobilization of Enzyme

OMVs were discovered over 50 years ago, and research on them has progressed significantly, particularly in modifying their functionality to expand their application potential. This review focuses on using OMVs as biocatalysts—particularly their functionalization with enzymes and their applications. Functionalizing OMVs for biocatalytic targets involves modified enzymes, with the immobilization of enzymes being the most common and relevant approach. Immobilized enzymes are stronger and more resistant to environmental changes than free enzymes in solution [20,21]. Additionally, OMVs can transport enzymes to distant targets in a protected state [22]. Therefore, the immobilization of enzymes enables the expansion of OMVs’ utility, while enhancing enzymes’ activity and stability.

There are several methods of enzyme immobilization, including depositing enzymes on solid matrices, adsorption on porous materials, immobilization via covalent bonds, affinity immobilization, and entrapment or encapsulation [20,23]. Each of these methods has its own advantages and disadvantages. Enzyme deposition on solid matrices and entrapment are the most commonly applied immobilization methods among these platforms. It is important to note that the chosen immobilization method significantly impacts the modified enzyme’s properties. For example, if the goal is to produce an enzyme with a thermally or mechanically stable surface, entrapment using OMVs is the appropriate immobilization method. On the other hand, enzyme deposition on solid matrices, such as enzyme display on the surface of OMVs, may be recommended if the substrate cannot penetrate the trapping matrix due to its limited properties. In relation to the above statements, the method of enzyme immobilization with the OMV platform is also closely related to the prospective applications of OMVs. In this review, we discuss recent research involving techniques pertaining to enzyme entrapment in OMVs and enzyme display on the surface of OMVs to elaborate on the method of enzyme immobilization with the OMV platform (Table 1).

### 2.1. Enzyme Display on OMV Surface

#### 2.1.1. Fusion with ClyA

The recent prevalence of OMV surface decoration may be traced back to one of the earliest investigations of Kim et al. [7]. That study functionalized the OMV surface using fusion binding with Cytolysin A (ClyA), a pore-forming hemolytic protein (Table 1). ClyA, initially identified in the *Escherichia coli* K-12 strain, is known to be one of the periplasmic proteins secreted along with the vesicles from the bacterial cell, suggesting its use in a vesicle transport mechanism in bacteria [6,28,29,30]. The expression of the fusion protein with ClyA outside the cell indicates that this porin can serve as an anchoring motif. While this study was one of the pioneer investigations into the use of OMVs as vaccines, the concept’s essence can also be applied to displaying enzymes, thereby broadening the use of OMVs as biocatalysts.

Kim et al. conducted experiments in which they fused several heterologous proteins to the ClyA toxin with N-terminus and C-terminus variations. The proteins β-lactamase (Bla), organophosphorus hydrolase (OPH), and β-galactosidase (LacZ) were coupled with ClyA to produce synthetic OMVs. Before combining ClyA with the enzymes, they fused it with GFP to assess the effect of fusion on the N- and C-termini. The combination of ClyA with GFP, Bla, and OPH, where ClyA was bound to the N-terminus fusion protein, showed a considerable increase in cell-surface and vesicle activity. On the other hand, Bla bound to the N-terminus of ClyA resulted in a significant decrease in activity. Similarly, ClyA bound to the C-terminus of OPH had no measurable effect on OPH activity. Given that the OPH substrate used in the study, paraoxon, is membrane impermeable, OPH bound outside the cell or vesicle was preferred [7]. In line with this hypothesis, Kim et al. also fused LacZ to the C-terminus of the ClyA anchor. Their research suggested that ClyA could serve as an anchoring motif for decorating the surface of OMVs with other proteins. In addition to ClyA, more than 60 membrane proteins have been identified as OMV anchoring proteins, including OmpA [9,31,32,33]. Further investigation of OMVs may uncover additional proteins with essential functions.

#### 2.1.2. Bioconjugation with SpyTag/SpyCatcher

The SpyTag/SpyCatcher system has emerged as a powerful tool for the covalent bioconjugation of proteins. SpyTag and SpyCatcher are derived from the CnaB2 domain of the fibronectin-binding protein FbaB of *Streptococcus pyogenes*. SpyTag is a small peptide consisting of 13 amino acids, while SpyCatcher is a larger protein fragment containing 116 residues. This system is based on the covalent peptide interactions between SpyTag and SpyCatcher protein pairs, which result in the formation of an irreversible isopeptide bond between lysine and aspartic acid residues. The isopeptide bond formation is spontaneous and occurs within minutes, without the need for specific pH or reaction temperature conditions. In addition, it is compatible with various buffers [34,35,36]. The simplicity and versatility of this system have made it a popular choice for protein ligation studies, including antibody targeting in vaccine development [37,38] and reporter conjugation in biosensor [39] applications.

SpyTag and SpyCatcher can be fused at either the N-terminus or the C-terminus. In addition to directly fusing the functional protein to the anchoring motif, the use of SpyTag or SpyCatcher to fuse with the C-terminus of the outer membrane protein is a way of functionalizing OMVs through surface display. One advantage of using SpyTag or SpyCatcher to fuse with the C-terminus of an outer membrane protein is the ability to display proteins that are challenging to transport out of the cell, such as large proteins or those with limited expression levels in the cytoplasm. To conjugate the fused protein with SpyTag or SpyCatcher, the only requirement is adding the fused protein to the buffer. For instance, the SpyTag/SpyCatcher system was used to display the virulence factor hemoglobin protease (Hbp) on the OMV platform to investigate optimal exposure of multiple antigenic sites to the immune system [37]. The Hbp–SpyTag display was achieved by fusing Hbp to the C-terminus of SpyTag, which enabled the stable attachment of various antigens to Hbp. In this case, SpyTag expressed far from the surface of OMVs provide broad access without steric hindrance for conjugation with its fusion partner, SpyCatcher, suggesting the potential for more stable attachment of various antigens to Hbp. The SpyTag/SpyCatcher system has also been employed to display the receptor binding domain (RBD) of the SARS-CoV-2 spike protein on the surface of OMVs. That study demonstrated the potential of the SpyTag/SpyCatcher system for displaying complex proteins and suggested its potential use for vaccine development against COVID-19 [38]. Although using the Spy toolbox for functionalizing OMVs as a biocatalyst is uncommon, the concept should inspire future development of OMVs for biocatalytic requirements. The SpyTag/SpyCatcher system offers a simple and efficient method for displaying proteins on the surface of OMVs (Table 1), providing a promising avenue for developing new biocatalysts.

Sometimes the functionalization of OMVs for biocatalyst purposes using the Spy system does not require engineering of the OMV surface. Instead, SpyTag is conjugated to the transmembrane porin protein OmpA at the internal, N-terminus, and C-terminus regions to create an entrapment platform for OMVs. For example, the phosphotriesterase (PTE) enzyme is fused with SpyCatcher, and the sequence leader torA is added to localize PTE-SpyCatcher expression in the periplasm. This allows the enzymes to be encapsulated within the OMVs [16,17]. This research demonstrates that applying the Spy tools is highly adaptable to achieve desired architectural structures and functions, and that it is not restricted to modifying OMV surfaces.

#### 2.1.3. The Utilization of the Ice-Nucleation Protein

The ice-nucleation protein (INP) is found in bacteria that actively nucleate ice, such as *Pseudomonas syringae* [40], *Pseudomonas fluorescens* [41], and *Erwinia herbicola* [42]. It can speed up the formation of ice crystals in supercooled water [43,44]. This protein comprises over 1200 amino acid residues and consists of three domains: the N-terminus domain, the central cylindrical repeating domain (CRD), and the C-terminus domain. The N-terminus domain is hydrophobic, while the C-terminus domain is hydrophilic. The N-terminus acts as a membrane anchor for the extracellularly expressed C-terminus; however, proteins fused to either or both termini are exposed on the cell surface. The CRD is a catalytic domain that contributes to the formation of ice crystals and is unrelated to anchoring motifs. However, it functions as a distance regulator between heterologous proteins and the cell surface [45,46].

Enzymes can be effectively immobilized on the outer membrane surface using the INP (Table 1). The INP has the ability to display the trivalent scaffold, Scaf3, on the bacteria cell surface by generating the INP-scaf3 fusion by engineering the episomal DNA [24,47]. Park et al. used the INP-scaf3 plasmid, containing three different cohesin domains, to immobilize multiple enzymes on the surface of OMVs simultaneously in the hyper-vesiculating mutant *E. coli* JC8031. This configuration allows the dockerin of each of the three different cellulases to interact with their respective cohesin partners, thereby providing a promising approach for the simultaneous and site-specific expression of multiple enzymes that are useful for cascade reactions [24].

Similarly, Su et al. reported using a different design to engineer the surface of OMVs using the INP. In their study, they fused the OPH enzyme directly to the INP in the pVLT33-INPOPH6 construct to immobilize the enzyme on the surface of OMVs. Additionally, they fused cellulose binding domains (CBD) with the *E. coli* lipidation sequence of major lipoprotein-outer membrane protein A (Lpp-OmpA) to form the pUCBD plasmid, in which Lpp-OmpA was used as an anchoring motif. This approach allows the two engineered proteins to be expressed outside of the cell, with the CBD providing an advantage in terms of OMV recovery and enabling the synthetic OMV-enzymes to be utilized multiple times [25].

Both studies demonstrated that the INP offers construction flexibility for displaying diverse functional proteins on OMV platforms and facilitates recovery and purification. Thus, this anchoring motif represents a promising tool for future biotechnological applications in enzyme immobilization.

### 2.2. OMV-Mediated Encapsulation

Encapsulation does not provide flexible interaction and high mass transfer between proteins and their environment. However, the encapsulation technique offers enzyme protection and increases enzyme stability by shielding enzymes from harsh environments, since the matrices will carry them in the lumen to travel long distances. The use of OMVs as the encapsulation matrices is more commonly applied in biomedicine for loading small molecules, such as pharmaceutical compounds [48,49]. However, this does not preclude its application for the biocatalytic functionalization of OMVs. Huang et al. categorized the functionalization of OMVs into (i) physical or chemical approaches and (ii) genetic engineering [50]. However, in this article, we do not discuss the functionalization of OMVs through a chemical approach—for instance, the 1-ethyl-3-[3-dimethylaminopropyl]-carbodiimide hydrochloride/*N*-hydroxysuccinimide (EDC/NHS) coupling reaction—as it is not a material encapsulation technique used for functionalized OMVs as biocatalysts. In addition, we examine the genetic-engineering-based functionalization of OMVs specifically for the encapsulation technique.

#### 2.2.1. Physical Functionalization

Incubation is the simplest way to introduce biomolecules or functional proteins into the lumen of OMVs. However, the loading efficiency is often uncontrolled, requiring large quantities of loading material to be incubated with the growth media. Alternatively, physical functionalization methods, such as electroporation, sonication, and extrusion, have been explored but require greater effort [50,51,52,53] For instance, electroporation has encapsulated gold nanoparticles (AuNPs) [51]. However, the use of this method for enzyme encapsulation and for utilizing OMVs as biocatalysts has yet to be documented, probably because it is difficult to prepare enzymes in large quantities. Another challenge is that the cargo must be delivered to the lumen while maintaining its enzymatic activity.

#### 2.2.2. Genetic Engineering Approach

Most OMVs used as biocatalysts are functionalized using genetic engineering approaches, such as the Spy toolbox mentioned earlier (Table 1). The idea of incorporating heterologous proteins into the lumen of OMVs was first explored by Kesty et al., who expressed GFP fused to the Tat signal peptide. This led to the detection of GFP in both the periplasm and OMVs, suggesting that OMVs partially enclose periplasmic contents and transport them outside the cell [26]. Later, the Spy system was developed, which utilized the SpyTag/SpyCatcher pair to bind the PTE enzyme to OmpA on the inner side of the OMV membrane [16,17,18]. In addition to bioconjugation with the Spy pair bound to the outer membrane protein OmpA, an alternative encapsulation method involves direct binding to the N-terminus of the lipidation sequence (Lpp) (Table 1), which has been demonstrated to hold the diisopropyl fluorophosphatase (DFPase) enzyme under the lipid bilayer [19].

Recent studies have shown the potential of signal peptides for localizing fatty acid-converting enzyme expression in the periplasm. While the Tat signal was previously used for this purpose, the enzyme is now secreted into the periplasm via the Sec pathway, guided by the PelB secretion signal peptide (PelBSS) from pectate lyase B of *Erwinia carotovora*. Fatty acid double bond hydratase from *Stenotrophomonas maltophilia* (SmOhyA) and photoactivated fatty acid decarboxylase from *Chlorella variabilis* NC64 A (CvFAP) were fused with PelBSS to allow for periplasmic expression, which was subsequently encased by OMVs [27]. Similarly, in another study, the β-lactamase enzyme encoded by the CMY-10 gene was secreted into the periplasm using PelBSS. Both studies employed a hyper-vesiculating *E. coli* mutant lacking the *tolA* and *tolR* genes [21]. The outer membrane protein serves as an anchoring motif for displaying enzymes or functional proteins extracellularly and as a holding motif for the loading material beneath the lipid bilayer during encapsulation. Moreover, since OMVs contain periplasmic contents, localizing loading material with signal peptides that can penetrate the inner membrane and accumulate in the periplasm significantly aids in immobilizing enzymes by encapsulating them using the OMV platform.

Overall, using signal peptides for enzyme localization and encapsulation within OMVs represents a promising approach for developing novel biocatalysts and drug delivery systems. While more research is needed to optimize the efficiency and specificity of this approach, it has the potential to revolutionize the field of biotechnology and to open new avenues for developing advanced therapeutics.

## 3. The Role of OMVs as Biocatalysts

OMVs have numerous applications in research [9,50], drug development [48,49,54], infection control [55], cancer treatment [48,56], bioimaging [57], and detection [58]. One of the most prevalent applications is in vaccine development [9,37,38,50]. However, the potential of OMVs as a biocatalyst platform is considered a valuable second-tier application. This literature review highlights the role of OMVs as biocatalysts through genetic engineering techniques for enzyme immobilization, as described previously. The use of OMVs as biocatalysts can be categorized into three main areas: (i) bioconversion, where OMVs act as a biocatalyst platform for chemical reactions; (ii) bioremediation, where OMVs assist in the degradation or hydrolysis of toxic compounds into environmentally safe materials; and (iii) biodegradation, where OMVs serve as a platform for degrading compounds other than those associated with environmental pollution. By exploring these applications, we hope to inspire further research into the potential of OMVs as a biocatalyst platform for various industrial and environmental processes.

### 3.1. Bioconversion

Recent studies have explored the potential of engineered OMVs to act as nanobioreactors for biocatalytic reactions. One of the most promising applications is the conversion of fatty acids into high-value compounds. The engineered OMVs containing SmOhyA and CvFAP enzymes catalyzed the conversion of oleic acid to 9-hydroxyheptadecane, a valuable ingredient in the cosmetics and pharmaceutical industries. The reaction involves a cascade reaction, starting with SmOhyA hydrating unsaturated fatty acids into hydroxy fatty acids, which are then converted into final products by CvFAP with the help of light. The specific biotransformation rates of oleic acid were reported to reach 8.0 × 10^−12^ μmol/min per OMV, demonstrating the high catalytic activity of OMVs. Moreover, CvFAP can directly decarboxylate the terminal carboxyl groups of unsaturated fatty acids into hydrocarbon compounds without a cascade reaction. Notably, the SmOhyA-encapsulating OMVs were found to be more stable than purified enzymes. The specific catalytic activity of OMVs was 100-fold greater than that of *E. coli* cells, indicating that OMVs derived from hyper-vesiculating *E. coli* can contain extremely concentrated enzymes [27].

Another example of using OMVs as biocatalysts is the display of CYP102A1 protein, a self-sufficient monooxygenase, on *E. coli*-derived OMVs to convert *para*-nitrophenoxydodecanoic acid or 12-pNCA substrate to *para*-nitrophenol, expanding the application of OMVs in the field of biosensors. Immobilization of CYP102A1 with OMVs demonstrates increased the stability and ease of biocatalyst use without the need for expensive enzyme purification [59]. Apparently, using OMVs as biocatalysts offers a promising platform for the efficient and sustainable production of high-value compounds. By utilizing genetic engineering techniques to immobilize enzymes within OMVs, researchers can harness the unique properties of these naturally occurring nanoparticles to create efficient and stable biocatalysts for a wide range of applications.

### 3.2. Bioremediation

Recent studies have shown that OMVs can be highly effective in bioremediation, where catalysts are often required. OMVs can be decorated with enzymes and proteins, such as Bla and OPH, to enhance their bioremediation potential. The success of displaying Bla and OPH on OMVs surface has been demonstrated by researchers investigating the anchoring ability of ClyA motifs. Synthetic Bla-containing OMVs have been shown to hydrolyze β-lactam antibiotics, while OMVs with OPH decoration can hydrolyze paraoxon [7]. Interestingly, enzymes fused to the C-terminus of ClyA showed 4-fold and 10-fold greater catalytic activity than those anchored to the N-terminus of ClyA for Bla and OPH, respectively, indicating that ClyA’s C-terminus fusion leads to the expression of enzymes outside the OMVs that are preferred by the substrate. In the same study, the differences between the enzymatic activity on the cell surface as a whole-cell biocatalyst and the enzymatic activity on OMVs were evaluated. Bla activity was relatively similar both on the cell surface and OMVs. Meanwhile, OPH showed a significant increase in activity when displayed on an OMVs surface compared to a whole-cell biocatalyst, with values of 81.63 × 10^3^ U/g total protein and 11.62 × 10^3^ U/A_600_, respectively, indicating that enzyme display on OMVs is preferred in this system [7].

In addition, researchers have immobilized OPH by anchoring it to the INP, which increased the enzyme’s stability and enhanced its degradation rate when tested with paraoxon as a substrate. The addition of CBD further enhanced the system, making these OPH-containing OMVs recoverable and retaining their activity even after 15 cycles [25]. The immobilization of Bla (encoded by the CMY-10 gene) was also reported to increase its stability and resistance to temperature and acid stress. The resulting Bla enzyme could degrade nitrocefin and meropenem 100-fold and 600-fold better, respectively, than whole-cell biocatalysts [21]. Another Bla-encoding gene, BT_4507 from certain *Bacteroides species*, was successfully immobilized to degrade cefotaxime [60]. Remarkably, the system developed by employing Bla can be utilized for the degradation of various antibiotics that may pollute the environment.

PTE is an enzyme that has been extensively studied for its ability to detoxify organophosphate compounds, which are used as pesticides and chemical warfare agents (CWA), including paraoxon. Researchers have recently found that OMVs can immobilize PTE and enhance its activity and stability [16,17,18]. When paraoxon penetrates the OMV via a transmembrane porin protein, it can reach PTEs within the vesicle and react with them. Compared to free PTE, PTE in OMVs has been found to be 100-fold more active and more resistant to multiple freeze–thaw cycles. This makes it an excellent candidate for use in environmental remediation, where the detoxification of organophosphate compounds is a critical concern. The effectiveness of PTE immobilized in OMVs has been tested on various solid surfaces, including glass, painted metal, and fabric, as well as in water samples from the environment. The promising results indicate that OMV-immobilized PTE can effectively hydrolyze organophosphate compounds on various surfaces and in different environmental conditions [16,17,18].

In a recent study, researchers have also shown that OMVs can be used to immobilize DFPase, another enzyme with organophosphate hydrolysis activity. The DFPase-OMV complex was found to be stable and effective in hydrolyzing diisopropyl fluorophosphate (DFP) and paraoxon. That study further emphasized the potential of OMVs for enzyme immobilization to preserve enzyme stability in different environments—in that case, by protecting the enzymes from temperatures as high as 80 °C and preventing catalytic activity loss [19].

Overall, OMVs have emerged as a promising tool for enzyme immobilization and have the potential to be used in a range of applications, including environmental remediation and biocatalysis. The enhanced stability, improved activity, and increased resistance to the environmental stress of enzymes immobilized in OMVs make them an attractive alternative to traditional immobilization techniques, which often involve artificial matrices or support structures. With further research and development, OMVs could offer an innovative solution to the growing problem of environmental pollution caused by various toxic substances, including those that are unsuitable for using living microorganisms.

### 3.3. Biomass Degradation

Researchers have developed a novel approach for immobilizing multiple enzymes on the surface of OMVs by constructing Scaf3 on the surface of OMVs. The first study to investigate this approach was aimed at assembling three cellulases for a cascade reaction to increase the efficiency of cellulose hydrolysis. Before incorporating the cohesion–dockerin interaction system, the localization of Scaf3 on the OMVs was confirmed using an immunofluorescence microscopy technique with a fusion tag probe. Subsequently, the researchers conjugated three cellulases (CelE, CelA, and BglA) to the surface of the OMVs. These enzymes are exoglucanase, endoglucanase, and β-glucosidase, respectively. The resulting platform significantly enhanced cellulose hydrolysis, increasing it by 23-fold compared to free enzymes. Moreover, the trivalent enzyme displayed on the OMV surface showed a 9-fold improvement in hydrolysis efficiency, compared to the same trivalent enzyme displayed on the yeast surface [24]. 

This approach has several advantages over other enzyme immobilization techniques. Using OMVs as a natural membrane-based scaffold for enzyme immobilization ensures stability, high activity, ease of manipulation, and the ability to incorporate multiple enzymes. Moreover, the cohesion–dockerin interaction system in that study enabled the specific binding of enzymes to the scaffold protein, making it easier to manipulate and optimize the enzymatic reaction. This technique could be applied in various fields, such as biocatalysis and biofuel production, where the cascade reaction of multiple enzymes is critical for efficient and cost-effective processes. The scaffold protein and OMVs can also be modified to target specific substrates or reactants, further increasing the specificity and efficiency of the enzymatic reactions.

## 4. Conclusions and Future Perspectives

Research on OMVs has rapidly advanced since their discovery. As miniature bacterial cells, OMVs have been found to stimulate immune responses, making them promising for developing vaccines and therapeutic agents. While the use of OMVs has primarily been explored in biomedicine, there has been increasing interest in their potential for other applications, especially for biocatalysis.

Although the number of studies of the catalytic potential of OMVs is less than the number of studies regarding its application in biomedicine, several studies have investigated this catalytic potential. One promising approach involves engineering techniques that enable the conjugation of OMVs with enzymes, which can be conveniently facilitated by genetic engineering. The use of OMVs for biocatalytic functions has several advantages, including protection against potential pathogen exposure during the application of the biocatalyst, high loading efficiency for encapsulating functional proteins located in the periplasm and the maintenance of catalytic function and enzyme stability.

However, the utility of OMVs still needs to be improved in certain respects, such as the costs and the labor-intensive purification process. Although challenging, the isolation and purification of outer-membrane vesicles are also being developed for industrial use, as documented in several granted patents [61,62]. Generally, OMVs are extracted using detergent to remove LPS and enhance vesicle release; then, purification is performed by ultracentrifugation and, optionally, followed by size-exclusion chromatography [61,62]. However, ultracentrifugation can result in low yields, due to the loss of certain desired components. Consequently, it is necessary to develop a more effective method for separating OMVs. To overcome this limitation, efforts have been made to develop strategies for recovering and reusing OMV biocatalysts. For example, the use of purification tags or domains on OMVs, such as CBD, has been explored to facilitate the recovery and reuse of OMV biocatalysts [25]. Future research could focus on developing new and more efficient methods for isolating and purifying OMVs.

Despite the progress that has been made, numerous applications of OMVs for biocatalysis can be explored in future research. For example, OMVs have shown promise in the development of biosensors and the production of fine chemicals. Additionally, there is potential for the use of OMVs in environmental remediation and the degradation of pollutants. Furthermore, OMVs have been shown to play a role in bacterial communication. Future research could focus on understanding the mechanisms behind this communication and how it could be exploited for medical or industrial purposes.

In conclusion, OMVs represent a promising platform for biocatalysis, due to their stability, activity, and ease of manipulation. Although there are limitations to their utility, the potential applications for OMVs are vast and future research is likely to focus on overcoming these obstacles and developing new and innovative ways to harness their unique properties for medical, industrial, and environmental purposes.

## Figures and Tables

**Table 1:** Methods for immobilizing enzymes using the OMV platform.

Enzyme Immobilization	Superiority	Deficiency	Detail Method/Illustration	Application	Ref.
***Surface display (enzyme display on OMVs surface)***					
Fusion with ClyA (and other OMVs’ anchoring motifs, such as OmpA)	Numerous outer membrane proteins can serve as anchoring motifs	Limited expression of proteins that are too large to be transported out of the cytoplasm	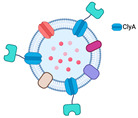	Degradation of paraoxon and antibiotics	[7]
Bioconjugation with SpyTag/SpyCatcher	Capable of displaying functional proteins that are challenging to export from the cell	SpyTag’s and SpyCatcher’s covalent bond is irreversible.	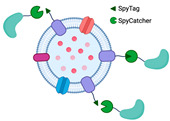	Little is known regarding the use of Spy systems to display enzymes on OMVs for biocatalyst purposes	*
Utilization of ice-nucleation protein (INP)	Facilitating assemblies with trivalent scaffolds is highly promising for simultaneously expressing multiple enzymes for cascade reactions	The size of INP is relatively bigger than Spycatcher.	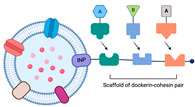	Enhanced glucose yield from cellulose degradation	[24,25]
			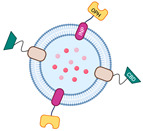	Paraoxon degradation to *para*-nitrophenol	[25]
***Encapsulation***					
Physical functionalization	Comparatively simple operation	Limited loading efficiency	- Incubation - Electroporation - Sonication - Extrusion	There has been no report on the use of this technique for enzyme entrapment in the OMVs lumen for biocatalytic purposes	
Genetic engineering	Improved enzyme stability and offers high loading efficiency	Limited interaction with the surrounding substrate	- The use of SpyTag/SpyCatcher 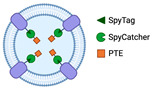	Decontamination of CWA, including paraoxon	[16,17,18]
			- Direct binding to Lpp-OmpA 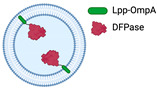	Hydrolyze DFP and paraoxon	[19]
			- Fusion with Tat secretion signal peptide	GFP entrapment in OMVs lumen (there have been no data for enzyme entrapment utilizes Tat signal for functionalization of OMVs as biocatalysts)	[26]
			- Fusion with Sec secretion signal peptide	Bioconversion of fatty acid	[27]

* We are currently working on this study in our laboratory. (All pictures are created with BioRender.com).

## Data Availability

Data sharing is not applicable to this article as no new data were created or analyzed in this study.
